# Active Assessment of Female Codling Moth, *Cydia pomonella* (L.), Mating Status Under Mating Disruption Technologies

**DOI:** 10.3390/insects17010041

**Published:** 2025-12-28

**Authors:** Alan Lee Knight, Michele Preti, Esteban Basoalto

**Affiliations:** 1 Instar Biologicals, Yakima, WA 98908, USA; 2 Independent Integrated Pest Management Consultant and Researcher, 48018 Faenza, Italy; 3 Facultad de Ciencias Agrarias y Alimentarias, Universidad Austral de Chile, Valdivia 5090000, Chile

**Keywords:** pear ester, (*E*,*Z*)-2,4-ethyl decadienoate, kairomones, female monitoring, unmated females, multiple matings, wild moths

## Abstract

Most assessments of sex pheromone-based mating disruption (MD) for codling moth (CM), *Cydia pomonella* (L.), have not included measurements of female mating. The recent development of a dual-sex lure (CM4K) combining ethyl (*E*,*Z*)-2,4-decadienoate, 4,8-dimethyl-1,3,7-nonatriene, pyranoid linalool oxide, and acetic acid has significantly increased the catch of female CM and made it easier to directly assess various MD programs. Samples of wild female CM populations using this lure were collected from 142 orchards, either untreated or treated with one of seven different dispenser systems for CM MD, from 2021 to 2022. The proportion of unmated females ranged from <0.20 to >0.80 with a median of 0.43 in both years. No differences in the mean proportion of unmated females were found between apple and pear. Moth immigration and supplemental insecticide sprays were hypothesized to be key factors affecting the variability within each MD tactic. Mating levels were significantly higher later in the season. More females were unmated in conventional compared with organic orchards. Means for females not mating among the CM MD programs varied over a 0.20 range, except for one. The exception occurred when two MD tactics (aerosol units plus hand-applied dispensers) were accidentally used together, and the proportion of unmated females was significantly higher.

## Simple Summary

Codling moth is a worldwide pest of apples and pears, as the larvae damage fruit by penetrating the skin and feeding on seeds in the core. Growers have widely adopted the use of sex pheromone dispensers to help manage codling moth in both crops. Integrated programs combine this behavioral disruption with insecticide applications. The success of these programs is evident by effective fruit protection, with the role of the dispensers evaluated largely by reductions in male moth catches. The actual mating status of the female moths has not been sufficiently evaluated with various types of dispensers. A new dual-sex non-pheromone lure can be used to ascertain the level of female mating. A two-year study (2021–2022) assessed different mating disruption programs across 142 orchards in Washington and Oregon, USA. The proportion of females mated varied widely among orchards in both years, but on average, the various dispensers achieved similar, moderate levels of mating disruption. The one exception was a combined aerosol and hand-applied dispenser program, which provided significantly greater mating disruption. Future studies need to evaluate a range of other intensive combination programs to assess their cost and success and determine whether insecticide usage could be further reduced with semiochemical-based management.

## 1. Introduction

The use of sex pheromone products to achieve mating disruption (MD) of codling moth (CM), *Cydia pomonella* (L.), has been heralded as a great success story in the implementation of stable Integrated Pest Management (IPM) programs in pome fruit worldwide [1,2]. Yet, an early review suggested the success of MD for CM was inconclusive and required a difficult set of preconditions to attain, i.e., a large area, a uniform canopy, a level slope, low pest density, and isolation of the orchard [3]. Subsequent technical improvements in dispensing systems, more effective monitoring, and adoption of MD over larger contiguous areas proved to be the keys to more effective outcomes [4,5,6,7,8]. Successes with MD for CM have primarily been documented through reductions in male catch in sex pheromone-baited traps and the attainment of acceptable levels of fruit injury when integrated with insecticide spray programs or in combination with sterile moth releases or enhanced cultural practices [9,10,11,12,13,14,15,16,17,18,19,20,21].

The early difficulty in developing effective CM MD involved technical formulation issues with stabilizing a conjugated diene pheromone under field conditions [22], as well as the fact that fruit injury by CM needs to be very low for fresh and export markets. Also, the early adoption of CM MD occurred during a difficult pest management period due to increasing worldwide loss of insecticide efficacy [23]. Recently, it was estimated that over 300,000 hectares of pome fruit and walnuts in the world are treated with CM MD [24]. This suggests that the early limitations have been solved or that pest managers have figured out how to integrate MD with other effective management strategies. Clearly, the available choices in insecticide selection are completely different today, and significant levels of resistance to these materials have only just begun to be reported in North America [25,26]. Unfortunately, no recent survey has reported the current usage of these new insecticides in combination with CM MD [27].

Surprisingly, one factor that has not been fully assessed in the current integrated management programs for CM is the contribution of MD. A recent review of 30 papers evaluating female CM mating success under MD surveyed 82 studies using both stationary assessments (N = 39), such as tethering and virgin female-baited traps, and active assessments (N = 43), such as oil-coated interception traps and sticky traps with pear ester-based lures [28]. The two methods used in each assessment type showed significant differences in the percentage reduction in females mating when compared with untreated blocks—stationary: 83 and 76% vs. active: 26 and 11%, respectively. The stationary methods measure the success of wild males mating with laboratory-reared females in a fixed position within the canopy. The active methods measure the mating of wild, dispersive females.

Studies included in the review reported the use of hand-applied dispensers at 800–1000 ha^−1^ [28]. Dispensers were either loaded with one or three components of the CM sex pheromone (PH) or just the major pheromone component, (*E*,*E*)-8,10-dodecadien-1-ol (codlemone), in combination with ethyl (*E*,*Z*)-2,4-decadienoate (pear ester) (PH/PE). Only a few studies used aerosols or sprayable MD, and these were not summarized. This review suggested that it remains unclear whether the overall success of integrated programs, including MD, could be due to the additive effects of reduced mating, delayed mating, reduced fecundity, and elevated natural control of adults and immature life stages [28]. The clear conclusion of this review was that the first focus should be to measure the “direct” effect of CM MD on female mating.

The identification of pear ester as a dual-sex adult attractant for CM has provided managers the ability to track female CM, and pear ester-based lures are widely used to establish action thresholds and to initiate phenology models [29,30]. Specifically, in MD-treated orchards, growers can effectively monitor both male and female CM by using the pheromone–kairomone combinational lure PHEROCON^®^ CM DA COMBO-P + AA (Trécé Inc., Adair, OK, USA), comprising codlemone, pear ester, and acetic acid [30]. The development of a new, more attractive kairomone blend (PHEROCON^®^ MEGALURE CM DUAL 4K, Trécé Inc., Adair, OK, USA; hereafter referred to as the CM4K lure) constructed with four non-pheromone components, including pear ester, has further increased our ability to collect female CM and measure female mating success [31,32,33,34,35,36].

Pear ester-based lures were found to have some bias for mated CM females when compared with concurrent catches on oil-coated clear interception traps placed in MD-treated orchards: 85 vs. 71% in the first flight and 91 vs. 84% in the second flight period [37,38]. Also, mated female CM were found to fly upwind more than virgins toward a pear ester source in a flight tunnel [39]. Despite this small bias, the more significant finding from the first study was that the majority of female CM captured in MD orchards were mated, and this was largely independent of moth population density in the treated orchard [38]. Similarly, the mating status of female CM caught with the CM4K lure has been consistent, with only an 18% higher proportion of unmated females caught in blocks treated with MD than without [35]. This poses the following question: If the majority of female CM under MD are mated, how can we conclude that MD is a successful technology that disrupts mating? Also, can growers adopt new MD programs that could be more effective?

Herein, we report the first extensive study of female CM mating success by surveying 142 pome fruit orchards treated with a variety of MD techniques, including hand-applied passive dispensers and aerosol dispensers, used at densities of 2.5–1000 ha^−1^ during 2021–2022 in Washington State and Oregon, USA. Orchards were monitored using delta traps baited with the CM4K lure, and data are presented separately for two seasonal flight periods in 2021–2022. These results suggest that CM MD programs, as currently implemented, can likely be improved.

## 2. Materials and Methods

### 2.1. Lures and Traps

Traps, liners, and lures were selected from commercially available monitoring systems for CM and were all provided by Trécé Inc., Adair, OK, USA. A proprietary binary lure was used in this study to sample male and female CM (PHEROCON^®^ MEGALURE CM DUAL 4K, Trécé Inc., Adair, OK, USA, referred to as CM4K). The CM4K lure comprised a black PVC matrix loaded with pear ester ((*E*,*Z*)-2,4-ethyl decadienoate), DMNT ((*E*)-4,8-dimethyl-1,3,7-nonatriene), and pyranoid linalool oxide (6-ethenyl-2,2,6-trimethyloxan-3-ol), used in combination with a white membrane cup dispenser loaded with acetic acid. Orange delta traps (PHEROCON^®^ DELTA VI, Trécé Inc., Adair, OK, USA) were attached to poles and placed in the canopy at ca. 3.0 m of height. Traps were placed >20 m from the borders and near the middle row of the plot. Traps were placed at a density of 1 per 2.0 ha. Lures were placed in the center of the sticky liner (CleanBrake^®^, Trécé Inc., Adair, OK, USA), and liners were replaced every 1–2 weeks. The binary lure was replaced after 8 weeks. Moths on liners were counted and sexed in the laboratory. All CM females sampled on a given date were dissected except when >30 females were collected, in which case only 30 were dissected. Female moths were dissected using a stereomicroscope to determine their mating status. Specifically, the level of female mating (unmated, single-mated, or multiple-mated) was assessed by dissecting the female abdomen and observing the *bursa copulatrix* to record the presence and number of spermatophores.

### 2.2. Mating Disruption Dispensers

Orchards were either left untreated or treated with MD dispensers for CM. Dispensers contained either codlemone alone or a blend of codlemone and pear ester. Seven types of MD programs based on label restrictions [40] were included in this study (Table 1). Two aerosol formulations and four hand-applied dispensers were considered according to the local growers’ practice. In addition, a new experimental hand-applied passive dispenser type, CPD (Concentrated Passive Dispenser), was constructed as a cluster of 8, 16, or 32 CIDETRAK^®^ CMDA COMBO MESO-A (Trécé Inc., Adair, OK, USA) dispensers clipped onto a 15 cm circular plastic hanger and applied at 10, 5, and 2.5 units ha^−1^, respectively, providing the same total amount of codlemone and pear ester per hectare as with the CIDETRAK^®^ CMDA COMBO MESO-A applied at 80 dispensers ha^−1^ (Trécé Inc., Adair, OK, USA). Only the 8-dispenser CPD placed at 10 units ha^−1^ was used in 2022. A previous proprietary study found no difference among CPD types with regard to moth catches or mating status (A.L.K., unpublished data). Finally, in four sites, the MESO or CPD dispensers were accidentally applied in combination with the aerosol MD, obtaining an eighth program (2X MD program) to be evaluated (Table 1). These four orchards were also treated with MD for two other tortricids, namely the Oriental fruit moth, *Grapholita molesta* (Busck), and the oblique banded leafroller, *Choristoneura rosaceana* (Harris), using additional aerosol units specifically for these two pests.

### 2.3. Seasonal CM Monitoring in Orchards Under Different Types of MD

Trapping studies were conducted during 2021 and 2022 in 75 and 67 pome fruit orchards, respectively (142 sites in total). Orchards were located in the upper and lower Yakima Valley (Tieton, Yakima, Wapato, and Sunnyside areas) in Washington State (USA) and in the Rogue Valley (Medford and Ashland areas) in Oregon (USA). Orchard size ranged from 2.0 to 10 ha; the median was 4.0 ha. Traps were deployed from 18 May to 13 September 2021 and from 23 May to 4 September 2022 to collect female CM to compare levels of mating across a range of MD tactics. Studies were conducted in organic and conventionally managed apple and pear orchards (Table 2). Orchards were representative of the pome fruit production area where the study was conducted, including ten apple cultivars (i.e., Red Delicious, Golden Delicious, Honeycrisp, Gala, Fuji, Ambrosia, Granny Smith, Envy, Cripps Pink, and Jazz) and seven pear cultivars (i.e., Bartlett, Red Bartlett, D’Anjou, Red Anjou, Bosc, Comice, and Seckel). Pear orchards were interplanted with 2–4 cultivars, and the most common apple block type was an interplanting of Red Delicious and Golden Delicious. A subset of orchards (N = 26) was monitored in both years, but these were not necessarily treated consecutively with the same MD program.

Two sampling approaches were used in the study. Several orchards were monitored with traps serviced every two weeks [28]. These data were summarized for the 1st flight (<1 July) or over the remainder of the season, which included the 2nd and potential 3rd flights [41]. The remaining orchards were monitored more inconsistently, and data could not be reliably subdivided into respective flights. Data for the entire season were summarized for all orchards (75 in 2021 and 67 in 2022, Table 2).

The categorization of orchards based on their CM MD program is summarized in Table 2. The use of CIDETRAK^®^ CMDA COMBO PP dispensers at rates between 500 and 840 ha^−1^ was the most frequently used MD program. This MD program was used in both organic and conventional orchards. NoMate CM Spiral and Isomate CM Flex dispensers and aerosol units were only used in organic orchards. The CIDETRAK^®^ CMDA COMBO MESO-A dispenser and three MESO cluster types (namely CPD) arrangements were used in both conventional and organic orchards. The combination of an aerosol plus MESO or CPD dispenser was accidentally applied to four organic apple orchards in 2021. A few orchards not treated with MD were sampled in 2021 (9 organic) and 2022 (6 conventional).

All dispensers were applied by the growers, except for the CPD programs. Aerosols were placed in a 35 m × 35 m grid starting 25 m from the edge of the orchards. Placement of hand-applied dispensers typically began <5 m from the edge of the orchard, and they were applied on every row at a spacing of 2 m to 3 m, depending on their density and row spacing. All orchards were sprayed by the grower with standard management and nutritional programs. Sprays applied for CM varied from 1 to 5 applications of diamide, spinosyn, and neonicotinoid chemistries. Organic orchards were treated with multiple applications of granulosis virus, horticultural oil, and 0–4 sprays of a spinosyn insecticide.

### 2.4. Statistical Analysis

Statistical analyses of the moth catches were performed with R software v. 4.4.2 [42]. Moth catch data were analyzed according to their distribution. Data normality was tested with Shapiro–Wilk’s test and Levene’s Test for Homogeneity of Variance. A linear model (*lm*) was used with normal data, while data that were not normally distributed were found to fit a Poisson distribution, and a generalized linear model (*glm*) was used. Akaike’s information criteria (*AIC*) and residuals were both used to select the fitted models in each analysis. A multiple comparison post hoc test was performed on the fitted models (*glht* function from the *multcomp* package), and Tukey’s HSD test (*p* < 0.05) was used to discriminate significant differences among treatments. Outputs reporting the F-statistic refer to normal data, while outputs reporting the X^2^ statistic refer to data not normally distributed. Treatments with a mean of zero catches were not included in analyses. Box and whisker plot figures were created using an R-specific function (*ggplot*). The female mating status data presented as box and whiskers plots were analyzed each year (2021 and 2022) with a linear mixed-effects model (*lmer*). The flight period (CM catches recorded before or after 1 July) and either the crop type (apple or pear) or the orchard management (conventional or organic) were set as explanatory variables, and their interaction was also tested in the model. Mating status data from each orchard were used in the analyses only when a sample of ≥5 CM females was dissected. Data are reported as mean values ± standard error (SE).

## 3. Results

**Figure 1:**
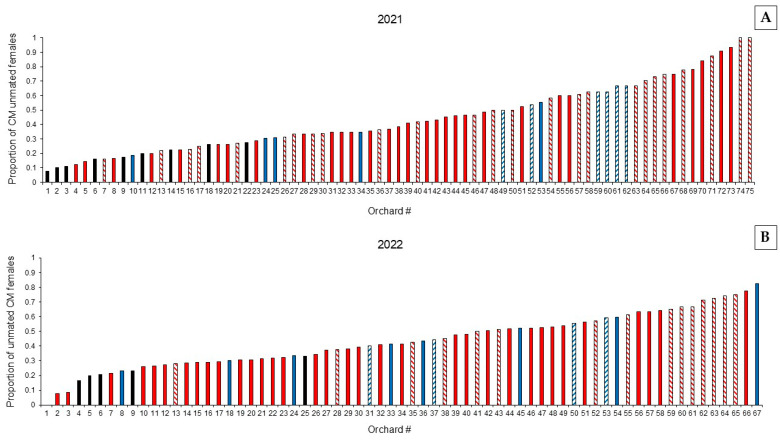
Proportion of unmated CM females collected during 2021 (**A**) and 2022 (**B**) in 75 and 67 pome fruit orchards, respectively, treated with different types of MD or not treated with MD (black columns). Red = apple orchard; blue = pear orchard; solid fill = organic management; pattern fill = conventional management. Orchard 1 in 2022 was untreated with MD and had no unmated females.

Over the two years of our study, 6126 CM females were dissected to determine their mating status. Interestingly, the mean proportion of unmated female CM varied widely across pome fruit orchards in both years (Figure 1). The proportion of unmated females across orchards ranged from <0.20 to >0.80. The median proportion of unmated female CM in orchards not treated with MD in 2021 was 0.17 across the season, compared to 0.43 in the MD-treated orchards. Data were similar in 2022, with the median proportion of unmated female CM of 0.21 in untreated orchards and again 0.43 in MD-treated orchards. 

**Figure 2:**
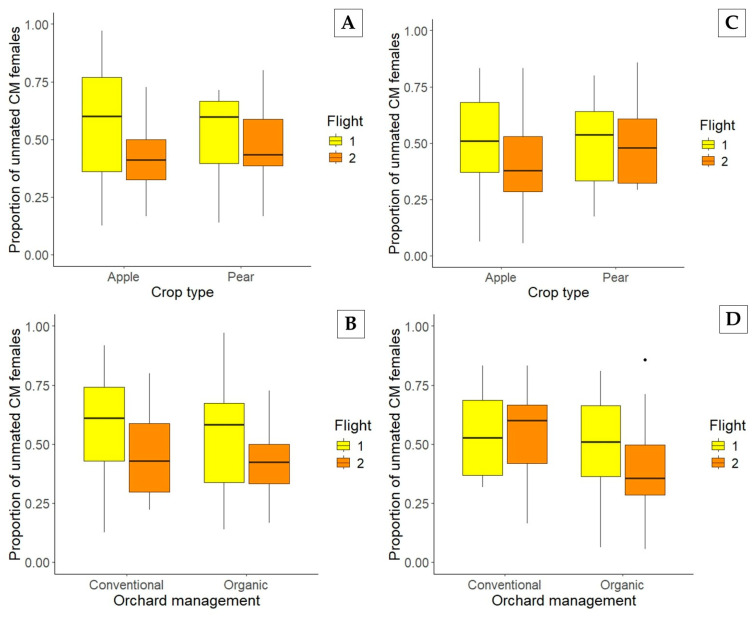
Proportion of unmated CM females caught in MD-treated orchards during the 1st and 2nd flights (yellow and orange, respectively), according to the crop type (**A**,**C**) and orchard management (**B**,**D**) in 2021 and 2022, respectively.

Factors such as crop type, orchard management, flight period, and MD treatment were found to have either a significant or nonsignificant effect on female CM mating in one or both years (Figure 2). In 2021, the proportion of unmated female CM in MD-treated orchards was impacted by the flight periods, regardless of crop type (Figure 2A) or orchard management (Figure 2B). Specifically, in 2021, in orchards treated with different MD programs, the proportion of unmated CM females was 0.56 ± 0.03 before 1 July and 0.43 ± 0.02 after 1 July, with a significant difference between flight periods (F_1,80_ = 10.335, *p* = 0.002). The difference between apple (0.50 ± 0.03) and pear (0.50 ± 0.04) was not significant (F_1,80_ = 0.001, *p* = 0.980), nor was the interaction between flight period and crop type (F_1,80_ = 2.111, *p* = 0.150). Orchard management in 2021 was not a significant factor influencing the proportion of unmated CM females: 0.53 ± 0.03 in conventional versus 0.48 ± 0.03 organic (F_1,80_ = 0.788, *p* = 0.377), and no interaction between flight period and management type was observed (F_1,80_ = 0.061, *p* = 0.806).

During 2022, similar patterns of these factors influencing female CM mating occurred (Figure 2). The proportions of unmated CM females early and late in the season were significantly different: 0.51 ± 0.03 before 1 July and 0.43 ± 0.03 after 1 July (F_1,87_ = 4.029, *p* = 0.048). Crop type was not a factor impacting the CM mating status in 2022 (Figure 2C), with no differences between apple and pear (0.46 ± 0.02 and 0.49 ± 0.04, respectively) (F_1,87_ = 0.461, *p* = 0.499) and no significant interaction between flight period and crop type (F_1,87_ = 0.648, *p* = 0.423). Nevertheless, one significant difference was found in the second year when comparing orchard management (Figure 2D). The proportion of unmated CM females was 0.54 ± 0.04 in conventional versus 0.44 ± 0.02 in organic orchards (F_1,87_ = 5.967, *p* = 0.017), while no significant interaction between flight period and management was observed (F_1,87_ = 1.605, *p* = 0.209).

Several significant differences were found among CM MD programs in 2021 (Table 3). Specifically: (*i*) The use of the CIDETRAK^®^ CMDA COMBO PP dispenser at 500–840 ha^−1^ in conventional orchards had less mating than the same treatment in organic orchards or in conventional orchards treated with the MESO or CPD dispensers; (*ii*) A 2X MD program including CIDETRAK^®^ CMDA COMBO MESO-A/CPD and the Semios aerosol applied in combination in four organic orchards had significantly higher proportions of unmated females than all other treatments tested in either year; (*iii*) The proportion of females mated more than once was significantly greater in the untreated orchards than under all MD programs (Table 3).

Data collected in 2022 were similar to those in 2021 (Table 3). For example, <0.20 of CM females in untreated orchards were unmated in both years. Three treatments had significantly greater proportions of unmated CM females than in the untreated orchards: NoMate CM, CIDETRAK^®^ CMDA COMBO MESO-A, and the experimental CPD dispensers. Three MD treatments were not significantly different from the untreated: Isomate CM Flex, CIDETRAK^®^ CMDA COMBO PP, and the Isomate CM Mist in organic orchards (Table 3). Similar to 2021, the proportion of multiple-mated female CM was significantly higher in the untreated orchards than in all MD treatments.

## 4. Discussion

CM has been categorized as a difficult tortricid to disrupt with sex pheromones, but MD has been widely adopted [24]. Disruption of male catch in sex pheromone-baited traps is clearly impacted by MD dispenser density and/or the total amount of pheromone released within the orchard [17]. Cumulative male moth catch is widely used to sample pest pressure; however, male catches are strongly influenced by lure selection and trap placement, which can vary widely among growers. The cost of increasing their standard rates of dispensers has likely prevented many growers from experimenting with ways to improve CM MD [43]. However, more importantly, their previous inability to assess the mating success of female CM under MD has precluded any quantitative research that could support changes in recommendations.

There are currently at least four companies with hand-applied dispensers, aerosols, and sprayable formulations for CM MD in the USA market [40]. Growers’ choice is dictated by previous success and recommendations from consultants and fieldmen. CM MD is only a part of the management program for CM; thus, growers can always choose to supplement their CM MD program with insecticides. Organic-approved insecticide choices are less effective than the conventional chemistry available for CM, and these growers would likely be the most receptive to improved CM MD programs. The serendipitous data recorded from four organic apple orchards in 2021 treated with two overlapping MD programs (aerosol + passive dispensers) hints that increased dispenser density and higher amounts of sex pheromone applied per hectare can significantly increase the level of CM MD. This approach needs to be more widely evaluated, and additional combinations should be assessed, such as mixes of aerosols, hand-applied dispensers, and sprayable formulations [44].

Several studies with CM have previously used >1000 dispensers ha^−1^ with dispenser spacings as low as 0.6 m [45,46,47]. Other studies have deployed multiple applications of higher densities of dispensers using sprayers or specialized equipment (fibers, flakes, clusters of microbeads) that release less pheromone per unit and are likely not comparable [17,48,49,50]. Finally, most of these experimental studies have been replicated only in small plots.

There has been little evidence that increasing dispenser rates above 1000 ha^−1^ can improve CM MD [45,51]. Grower adoption of areawide programs for CM in the 1990s required steep subsidies from governments [6,8]. Growers have experimented with higher rates of hand-applied dispensers, often on borders and considering the dominant wind direction and possible hotspots with high levels of infestation [1,52]. These areas can also be treated with additional insecticide sprays instead of more dispensers [10,15,53]. Increasing point sources from 2.5 to 5.0 ha^−1^ with aerosols is twice as expensive but is not a significant labor issue [54]. Microencapsulated formulations can be added to regular applications of pesticides [44]. Increasing labor costs and the reduced availability of the workforce when applying hand-applied dispensers at rates > 1000 ha^−1^ over the entire orchard could represent a strong disincentive. However, without a direct method to measure mating levels, there has not been any support to change the CM MD program, and most growers simply follow the status quo. But a combined program using either sprayables or aerosols plus hand-applied dispensers would likely be an intermediate expense and could be a more effective program.

Our survey of CM female mating status in 142 apple and pear orchards suggests that there are many situations where CM MD is not performing well for growers, specifically when more than half of the female CM in the orchard are mated. The data collected in 2021, combining aerosols and hand-applied dispensers, shows that CM MD can perform at a much higher level. We suggest that growers with significant CM management issues could adopt more intense MD programs. These can be either increasing dispenser rates or combining more than one tactic, such as aerosol or sprayables plus hand-applied dispensers [44]. The effectiveness of these new programs can only be ascertained via sampling significant reductions in female mating, and not the reduction in male catch in traps [28].

We suggest that adopting a standard protocol could be used to assess CM mating success across MD programs. For example, quantifying female mating following releases of internally marked, sterilized, unmated CM adults would help to minimize the potential impact of variable immigration levels (Okanagan–Kootenay Sterile Moth Program in Osoyoos, British Columbia) [55]. Moths are readily available to growers over most of the USA and have been shipped to other countries [21,56,57], or new colonies have been established to produce sterilized moths [58]. Sterilized Canadian moths were previously used to measure female CM mating under exclusion netting, where moth immigration was prevented [59].

Two inputs in addition to MD are known to impact the mating status of CM females, and their variable use may have contributed to the wide range found in the proportion of unmated female CM. The first is the topical and residual exposure to selected insecticides used in conventional pome fruits. Data collected in 2021 in orchards treated with CIDETRAK^®^ CMDA COMBO PP dispenser supports this possible effect, as significantly fewer females were mated in conventional compared with organic programs despite using the same CM MD program.

The anthranilic diamide, chlorantraniliprole, is widely used in conventional tree fruit management, primarily during one generation of CM if growers are following the resistance management label recommendations [40]. Previously, we found that residual exposure to chlorantraniliprole impacts female CM mating levels in both laboratory and field trials [60]. Similar results with chlorantraniliprole were later found with other lepidopteran pests [61,62]. Interestingly, season-long use of chlorantraniliprole resulted in greater mating reduction in wild female CM than the use of sex pheromone dispensers over a two-year study (28.5 vs. 7.5%) [60]. In the present study, 1–3 chlorantraniliprole sprays were used to manage CM in conventional orchards.

Sublethal effects of other insecticides that are used in pome fruits could also impact female CM mating. The ecdysone agonist, methoxyfenozide, timed for peak oviposition, can impact adult CM by reducing fecundity and fertility and male flight, but an effect on mating has not been reported [63]. However, none of the orchards in our study used this insecticide. Neonicotinoid insecticides, including acetamiprid, which is used in tree fruits for CM, can have significant sublethal effects on pests, including adult longevity, fecundity, and fertility [64]. Yet, few studies of sublethal activity of neonicotinoid insecticides on lepidopterans have been published and do not include any data on mating [65]. In our study, acetamiprid was only used in a few conventional orchards and only during the second half of the season, when female mating was actually higher than during the first flight.

A second type of spray material that can affect female CM mating—but that is more typically used to enhance insecticides—is a microencapsulated pear ester formulation, CIDETRAK^®^ DA MEC^®^ (hereafter named as PE MEC, Trécé Inc., Adair, OK, USA) [66,67]. PE MEC is also organically registered and is commonly used in combination with a granulosis virus [68,69]. Previously, the use of PE MEC was found to have a limited impact on the mating success of females when used alone or in combination with a microencapsulated sex pheromone spray [70]. However, the addition of PE MEC to the sprayable sex pheromone significantly decreased the proportion of multiple-mated CM females in one out of two years in walnuts and reduced nut injury [63]. Other studies have found that PE MEC significantly reduced levels of CM fruit injury when used in combination with PH or PH/PE dispensers for MD [58,59]. PE MEC was used with chlorantraniliprole in some conventional orchards and with granulosis virus in most organic orchards in our two-year study.

A final consideration for growers before they increase their cost for CM MD is how they can measure the potential benefits achieved by further reducing female mating. Obviously, growers and managers believe that their current use of CM MD has a value that offsets its cost, ca. USD 250 ha^−1^ [43]. Adopting CM MD is thought to reduce cullage and unnecessary insecticide sprays [6,7,8]. Neither the value of preventing an average of 30% of female CM from mating under typical MD programs [35] nor the value of bumping up MD by an additional <30–40% is currently known. Creating a simple economic–biological model suggests that the value of MD is heavily dependent on both the prevention of fruit injury and the value of the cultivar [35]. Growers with orchards of more valuable cultivars or organic certification have the strongest incentive to improve CM MD.

## 5. Conclusions

The discovery of the CM4K lure allows researchers and managers to ascertain the relative effectiveness of their CM MD program. We used traps with this lure to monitor the female CM mating status in 142 apple and pear orchards during 2021–2022. The proportion of unmated female CM was similar in both years and did not differ between apple and pear. Fewer female CM were unmated in just one of the two years in organic versus conventional orchards. The proportion of mating was higher in the second half of the season. Seven different CM MD programs had similar levels of unmated females in one or both years. The major exception occurred in four organic apple orchards in 2021 that used a combined aerosol plus hand-applied dispenser program. The observation of an extremely high proportion of unmated females (close to 0.90) in this program provides impetus to further investigate combined MD tactics to improve the disruption of matings for CM. We suggest that a standardized protocol could be used to minimize the differential impact of moth immigration and allow a more rigorous assessment of CM MD programs. Future studies could also clarify how supplemental spray programs and moth immigration impact female mating status to further implement MD technologies for CM.

## Figures and Tables

**Table 1:** Summary of CM MD dispensers used in growers’ orchards, 2021–2022.

CM MD Programs	Type of CM MD	Manufacturer	Active Ingredients Loading	Dispenser Rate ha^−1^
1	Semios CM Eco Aerosol	Semios BIO Technologies Inc., Vancouver, BC, Canada	55.4 g codlemone	2.5
2	Isomate CM Mist	Pacific Biocontrol Corp., Vancouver, WA, USA	23.6 g codlemone	2.5
3	NoMate CM Spiral	Scentry Biologicals Inc., Billings, MT, USA	135 g codlemone	1000
4	Isomate CM Flex	Pacific Biocontrol Corp., Vancouver, WA, USA	95.5 g codlemone + 62 mg of 1-dodecanol and 1-tetradecanol	1000
5	CIDETRAK CMDA COMBO PP	Trécé Inc., Adair, OK, USA	90 g codlemone 60 g pear ester	500–840
6	CIDETRAK CMDA COMBO MESO-A	Trécé Inc., Adair, OK, USA	850 g codlemone 500 g pear ester	80
7	Experimental CPD	Trécé Inc., Adair, OK, USA	850 g codlemone 500 g pear ester	2.5–10
8	Semios CM Eco Aerosol + MESO-A/CPD	Semios BIO Technologies Inc., Vancouver, BC, Canada + Trécé Inc., Adair, OK, USA	55.4 g codlemone + 850 g codlemone 500 g pear ester	2.5+ 2.5–80

**Table 2:** Summary of 142 orchards where the female CM mating status was sampled during 2021–2022, Washington State and Oregon, USA.

Year	Crop	Management	Types of CM MD ^a^	Number of Orchards	Monitored Both Flights ^b^
2021	Apple	Conventional	CMDA PP	21	15
			CMDA MESO	1	1
			exp. CPD	3	0
	Apple	Organic	no MD	5	0
			CMDA PP	14	14
			CMDA MESO	3	0
			exp. CPD	9	0
			Semios Aerosol + CPD/MESO (2X MD)	4	4
	Pear	Conventional	CMDA PP	6	6
	Pear	Organic	no MD	4	0
			CMDA PP	5	5
**Total**				**75**	**45**
2022	Apple	Conventional	no MD	3	0
			CMDA MESO	6	6
			exp. CPD	9	9
	Apple	Organic	CMDA PP	9	8
			CMDA MESO	3	2
			exp. CPD	2	0
			Isomate Aerosol	7	7
			Isomate CM Flex	6	5
			NoMate CM Spiral	7	5
	Pear	Conventional	no MD	3	0
			CMDA MESO	2	2
			exp. CPD	2	2
	Pear	Organic	CMDA PP	6	4
			NoMate CM Spiral	2	2
**Total**				**67**	**52**

^a^ Different types of mating disruption dispensers were evaluated, including CIDETRAK^®^ CMDA COMBO PP (CMDA PP); CIDETRAK^®^ CMDA COMBO MESO-A (CMDA MESO); CIDETRAK^®^ CMDA COMBO MESO-A arranged in clusters to form an experimental CPD (exp. CPD); Semios CM ECO (Semios Aerosol); Isomate CM Mist (Isomate Aerosol); Isomate CM Flex; and NoMate CM Spiral. Some orchards were not treated with mating disruption (no MD). Four orchards in 2021 were treated with Semios Aerosol plus CPD/Meso dispensers (2X MD). ^b^ Includes orchards sampled biweekly during both time periods.

**Table 3:** Summary of female codling moths caught in orchards (apple and pear) monitored during 2021–2022 under various forms of mating disruption or untreated in Washington State and Oregon (USA).

Year	Treatment, Dispensers ha^−1^, Management	Number of Orchards	Number of Dissected Females	Mean Values (±SE) ^a^	
				Proportion of Unmated Females	Proportion of Multiple-Mated Females
2021	Untreated, organic	9	350	0.18 ± 0.02 d	0.12 ± 0.03 a
	CIDETRAK CMDA PP, 500–840, conventional	25	554	0.54 ± 0.04 b	0.02 ± 0.01 b
	CIDETRAK CMDA PP, 500–840, organic	19	1448	0.39 ± 0.02 c	0.03 ± 0.01 b
	CIDETRAK CMDA MESO-A, 80, conventional and organic	4	189	0.32 ± 0.04 bcd	0.03 ± 0.01 b
	Experimental CPD, 2.5–10, conventional and organic	11	402	0.28 ± 0.04 cd	0.05 ± 0.02 b
	CIDETRAK CMDA MESO/CPD, 80/2.5–10 + Semios Aerosol, 2.5, organic	4	145	0.87 ± 0.03 a	0.00 ± 0.00
			Statistic	*X*^2^ = 97.09, *p* < 0.001	*X*^2^ = 51.33, *p* < 0.001
2022	Untreated, conventional	6	78	0.19 ± 0.04 c	0.10 ± 0.03 a
	NoMate CM, 1000, organic	9	704	0.47 ± 0.06 ab	0.04 ± 0.01 b
	Isomate CM Flex, 1000, organic	6	352	0.33 ± 0.09 bc	0.01 ± 0.01 b
	CIDETRAK CMDA PP, 500–840, organic	15	711	0.38 ± 0.03 bc	0.02 ± 0.01 b
	CIDETRAK CMDA MESO-A, 80, conventional and organic	10	571	0.46 ± 0.05 ab	0.03 ± 0.02 b
	Experimental CPD, 10, conventional and organic	12	319	0.58 ± 0.05 a	0.01 ± 0.01 b
	Isomate CM Mist Aerosol, 2.5, organic	7	303	0.45 ± 0.06 abc	0.03 ± 0.01 b
			Statistic	F_6,58_ = 5.15, *p* < 0.001	*X*^2^ = 55.48, *p* < 0.001

Orchards with traps catching overall < 5 females, such as treatments with mean values equal to zero, were not included in the analyses. ^a^ Mean values within the same column within each year, followed by different letters, are significantly different (Tukey test, *p* < 0.05).

## Data Availability

Data supporting the results are openly available in the public repository Zenodo at https://doi.org/10.5281/zenodo.17512294 (accessed on 23 December 2025).
